# The Role of the Endocannabinoids in Suppression of the Hypothalamic-pituitary-adrenal Axis Activity by Doxepin

**Published:** 2011

**Authors:** Parichehr Hassanzadeh, Anna Hassanzadeh

**Affiliations:** 1 *Research Centre for Gastroenterology and Liver Diseases, Shahid Beheshti University of Medical Sciences, Tehran, Iran*; 2 *Neuropsychopharmacology Research Center, AJA University of Medical Sciences, Tehran, Iran*; 3 *Department of Molecular Biology, Faculty of Molecular & Cellular Sciences, Islamic Azad University, Parand, Iran*

**Keywords:** Brain, Doxepin, Endocannabinoid system, HPA axis, Rat

## Abstract

**Objective(s):**

The mechanism(s) by which antidepressants regulate the hypothalamic-pituitary-adrenal (HPA) axis remain elusive. The endocannabinoid system (eCBs) which exhibits antidepressant potential, appears to regulate the HPA axis activity. Therefore, we aimed to investigate the role of the eCBs in the action of doxepin including its effect on the HPA axis.

**Materials and Methods:**

Male Wistar rats received acute and four-week intraperitoneal injections of doxepin (3, 5, and 10 mg/kg) or its vehicle (0.9% saline). One hr after the last injection, animals were exposed to a 5 min swim stress session. In other cohorts of animals, the CB_1_ receptor antagonist AM251 (0.25, 0.5, and 1mg/kg) was injected 30 min before the administration of doxepin. Plasma corticosterone concentration was measured by enzyme-immunoassay at 45 min following stressing. 1, 5, and 12 hr after the last injection of doxepin, the contents of endocacannabinoids (anandamide and 2-arachidonylglycerol) within the lipid extracts of the prefrontal cortex, amygdala, hippocampus, and hypothalamus were determined using isotope-dilution liquid chromatography-mass spectrometry.

**Results:**

Chronic treatment with doxepin (10 mg/kg) significantly reduced the secretion of corticosterone due to 5 min exposure to swim stress. Acute administration of doxepin evoked no effect. Pre-application of AM251 (1 mg/kg) abolished the ability of doxepin to reduce corticosterone secretion. Chronic administration of doxepin (10 mg/kg) led to a significant elevation of the endocannabinoids in the examined brain regions.

**Conclusion:**

It appears that doxepin exerts its effects, at least in part, through activation of the eCBs and the CB_1_ cannabinoid receptors play a major role in this regard.

## Introduction

Since their introduction, tricyclic antidepressants (TCAs) have mostly been used in depression, a widespread illness that is characterized by an array of disturbances in emotional, cognitive, and neurovegetative processes (1,2). There is general agreement that over-activity of the hypothalamic-pituitary-adrenal (HPA) system is a typical neuroendocrine sign of moderate to severe depression (3). It has been postulated that antidepressants may exert their therapeutic effects, at least partly, through their actions on the HPA system. They up-regulate central glucocorticoid receptors, thereby dampening the HPA-system activity (4,5). Understanding of the nature and causes of depression has evolved over the centuries; however, many aspects of depression including its complex neurobiology remain incompletely understood and are the subject of discussion and research (2,4). Furthermore, the mechanism(s) by which antidepressants regulate the HPA axis activity remain elusive. In recent years, the eCBs and its regulatory functions both in the central and peripheral nervous systems has triggered an exponential growth of studies. The eCBs is engaged in a plethora of physiological functions and has opened up new strategies in the treatment of pain, obesity, neurological diseases such as multiple sclerosis, and emotional disturbances (6-11). Dysregulation or malfunctioning of the eCBs may contribute to the aetiology of anxiety-related disorders and certain symptoms of depression (12,13). According to the reports, enzymes involved in the synthesis or degradation of the eCB ligands and CB_1_ cannabinoid receptors are prevalent throughout the neuroanatomical structures and circuits implicated in depression (14,15). In an animal model of depression, enhancement of the CB_1_ receptor activity exhibited antidepressant-like response (16). In addition to a critical role for the eCBs in the regulation of HPA axis activity (17-19), the location of the CB_1_ cannabinoid receptors on glutamatergic terminals in the paraventricular nucleus of the hypothalamus (PVN) and activation of the corticotrophin releasing factor (CRF) neurosecretory cells have been suggested (20,21). It is reminded that the levels of CRF and cortisol are increased due to depression (22,23), while, the ability of these hormones to exert negative feedback on the HPA axis activity is suppressed (3-5). Therefore, normalization of glucocorticoid feedback and hypersecretion appears to be associated with better prognosis. Since the eCBs appears to regulate the HPA axis activity and is implicated in both the pathophysiology and treatment of depression, therefore, we aimed to investigate the contribution of the eCBs to the ability of a TCA to suppress stress-induced activation of the HPA axis. We selected doxepin, a widely used TCA in the treatment of major depression and other psychiatric disorders with strong anxiolytic action that makes it useful in the perioperative period (24). However, in contrast to the prototype TCAs such as imipramine, less attention has been paid to doxepin in the mechanistic studies. To further explore the implication of the eCB transmission in the action of doxepin, we measured the levels of two major endocannabinoids, anandamide and 2-arachidonylglycerol (2-AG), in the brain regions crucial for the regulation of emotionality and stress including the prefrontal cortex, hippocampus, hypothalamus, and amygdala (25-27).

## Materials and Methods

***Animals***

Male Wistar rats weighing 250-280 g were purchased from the Pasteur Institute () and housed in pairs under standard conditions on a 12- hr light/dark cycle. Food pellets and water were available *ad libitum.* Experiments began after at least 1 week of habituation to the housing conditions. The procedures were approved by the local Ethics Committee of AJA University of Medical Sciences, . 

***Neuroendocrine studies***

Animals received acute or chronic (28 days) once-a-day intraperitoneal (i.p.) injections of doxepin hydrochloride (Sigma Aldrich, Germany) at doses of 3, 5, and 10 mg/kg (24) which was dissolved in 0.9% saline (n= 6/group). Injections were given in a volume of 1 ml/kg between 9.00 and 10:00 a.m. Control groups received an equivalent amount of saline (n= 6/group). One hr after the last injection of doxepin or its vehicle, animals were exposed to a 5 min swim stress session which was performed in a cylindrical Plexiglas container, filled to a height of 30 cm with water at 21 °C (28). Forty five min after the stress, animals were subjected to a tail bleed to obtain blood for analysis of plasma corticosterone. For plasma collection, blood samples (200 µl) were collected in plastic tubes containing 10 µl EDTA (4%). Fresh blood was kept on ice and centrifuged for 15 min at 4000×g (4 °C). The plasma samples were stored at -80 °C until the time of the assay. Corticosterone levels were measured by enzyme-immunoassay using a commercial kit (Immunodiagnostic Systems Ltd., ). All samples were run in duplicate and kit calibrators and controls were included in each analysis. Absorbance was measured at 450 nm with a reference wave length of 650 nm in an ELISA microplate reader. The sensitivity of the assay was 5 ng/ml and the intra- and inter-assay coefficients of variation were 4.4% and 6.5%, respectively. In case of a significant alteration in the stress-induced plasma corticosterone level by doxepin, the cannabinoid CB_1_ receptor antagonist AM251 (Tocris Bioscience, UK) was dissolved in Tween 80 (Sigma Aldrich, Germany), dimethyl sulfoxide (Sigma Aldrich, Germany), and 0.9% saline (1:1:8) and injected i.p. at doses of 0.25, 0.5, or 1 mg/kg 30 min prior to the administration of doxepin in order to evaluate the role the CB_1_ receptors in this regard. In parallel, there were animal groups receiving AM251 alone. Groups of animals under no stress condition were also considered in order to evaluate the effects of treatment conditions on basal activity of the HPA axis. 

***Biochemical studies***

One, 5, and 12 hr after the last injection in acute or chronic administration of doxepin or its vehicle (n= 6/group), animals were rapidly decapitated and the brain of each animal was quickly and carefully removed from the skull (29,30). The prefrontal cortex, amygdala, hippocampus, and hypothalamus were dissected out on a frozen pad taken from a -80 °C freezer using the Paxinos and Watson atlas (1986) for morphological orientation. All tissues were weighed and immediately frozen in liquid nitrogen and stored at -80 °C for further quantification of endocannabinoids. In the extraction of endocannabinoids from the frozen tissues, each tissue sample was homogenized in 5 vol of chloroform/methanol/Tris-HCl 50 mM (2:1:1) containing 500 pmol of d_8_-anandamide and d_8_-2-AG. Deuterated standards were synthesized from d_8_-arachidonic acid and ethanolamine or glycerol. Homogenates were centrifuged at 13,000×*g *for 16 min (4 °C), the aqueous phase plus debris were collected and extracted twice with 1 vol of chloroform. The organic phases from the three extractions were pooled and the organic solvents eliminated in a rotating evaporator. Then, the lyophilized samples were stored at -80 °C under nitrogen atmosphere until analysis. In order to analyze the eCB contents, the lyophilized extracts were re-suspended in chloroform/methanol 99:1 (v/v), then, the solutions were purified by open bed chromatography on silica. Fractions eluted with chloroform/methanol 9:1 (v/v) (containing anandamide and 2-AG) were collected and the excess solvent eliminated with a rotating evaporator and aliquots analyzed by normal phase high-pressure liquid chromatography (NP-HPLC) carried out using a semipreparative silica column (Spherisorb S5W, Phase Sep, Queensferry, CLWYD, UK) eluted with a 40-min linear gradient from 9:1 to 8:2 (v/v) of n-hexane/2-propanol (flow rate= 2 ml/min). These elution conditions allow the separation of 1(3)- and 2-acylglycerols (retention time of 18 and 20 min, respectively) from N-acylethanolamines (retention time= 26-27 min). NP-HPLC fractions from 17 to 22 min and from 24 to 28 min were pooled, the solvent evaporated in a Speed-vac, and the components derivatized with 20 µl N-methyl-N-trimethylsilyl-trifluoroacetamide and 1% trimethylchloro-sylane for 2 hr at room temperature and analyzed by gas chromatography/mass spectrometry (GC-MS). Monoacylglycerols or N-acylethanolamines with different fatty acid chains were also separated. MS detection was carried out in the selected ion monitoring mode using m/z values of 427 and 419 (molecular ions for deuterated and undeuterated anandamide), 412 and 404 (loss of 15 mass units from deuterated and undeuterated anandamide), 530 and 522 (molecular ions for deuterated and undeuterated 2-AG), and 515 and 507 (loss of 15 mass units from deuterated and undeuterated 2-AG). The area ratios between signals of deuterated and undeuterated anandamide varied linearly with varying amounts of undeuterated anandamide (20 pmol-20 nmol). The same was applied to the area ratios between signals of deuterated and undeuterated 2-AG in the 100 pmol-20 nmol interval. The levels of anandamide and 2-AG in unknown samples were calculated on the basis of their area ratios with the internal deuterated standard signal areas. Two GC-MS peaks for both deuterated and undeuterated mono-arachidonoylglycerol were found corresponding to 2-AG and 1(3)-AG. The amounts of 2-AG were calculated by adding the amounts of the two isomers (29-33). The amounts of endocannabinoids were expressed as pmol or nmol per gram of wet tissue extracted. The measurements were performed in duplicate and analyzed by an investigator blind to the experimental set-up.

***Statistical analysis***

The Kolmogorov-Smirnov test was used to verify normal distribution of the experimental data. One-way analysis of variance (ANOVA) and two-way ANOVA followed by the Tukey’s test (StatView 5 software; SAS Institute Inc., ) were used respectively for data analysis in the neuroendocrine and biochemical studies. Data are presented as mean±SEM (6 animals per group). The level of significance was set at *P*< 0.05. 

## Results

***Effect of doxepin and AM251 on plasma corticosterone levels under basal or stress conditions***

Post hoc analysis revealed that exposure to the swim stress significantly increased plasma corticosterone level (Table 1, *P*< 0.001). 28-day treatment with doxepin (10 mg/kg) led to a significant reduction in stress-induced plasma corticosterone level as compared to vehicle-treated stressed rats (Table 1, *P*< 0.05). Acute treatment with doxepin at all doses tested or repeated injections at doses of 3 or 5 mg/kg evoked no effect (data not shown). Administration of doxepin in animals that had not been exposed to the stressor did not alter corticosterone level (Table 1, *P*> 0.05). Daily injections of AM251 (1 mg/kg) prior to the treatment with doxepin abolished the ability of doxepin to reduce corticosterone secretion as compared to vehicle-treated stressed rats (Table 1, *P*> 0.05), whereas, the CB_1_ receptor antagonist at doses of 0.25 or 0.5 mg/kg did not show any preventive effect (Table 1, *P*< 0.05). Administration of AM251 alone did not affect aplasma (plasma) corticosterone concentration in non-stressed or stressed conditions (Table 1, *P*> 0.05). 

***Effect of doxepin on brain regional levels of endocannabinoids***

Acute administration of all doses tested or chronic treatment with 3 or 5 mg/kg doxepin did not affect the eCB contents in any brain region examined (data not shown), while, four-week administration of 10 mg/kg doxepin resulted in a significant elevation of both anandamide and 2-AG levels in all brain structures tested at 1 hr from the last injection (Table 2, *P*< 0.05 or *P*< 0.01 vs. control groups) that lasted for up to 5 and 12 hr (not shown).

***The CB1 receptor antagonist dose-dependently blocks doxepin-induced elevation of endocannabinoids***

Daily injections of AM251 (1 mg/kg) prior to the treatment with doxepin (10 mg/kg), prevented the augmentation of brain regional levels of endocannabinoids (Table 2, *P*> 0.05). Pre-application of AM251 at doses of 0.25 or 0.5 mg/kg did not show a preventive effect (Table 2, *P*< 0.05).

**Table 1.** Effect of the chronic administration of doxepin and AM251 on plasma corticosterone levels under basal or stress conditions.

Plasma corticosterone levels (ng/ml) following the 28-day treatment period	No stress	Swim stress
Vehicle of doxepin	89.76±8.76	468.23±18.08 ***
Vehicle of AM251	90.55±8.02	459.51±18.98 ***
Doxepin (10 mg/kg)	97.32±8.46	378.26±17.68 *** †
AM251 (1 mg/kg)	96.22±8.35	473.06±19.98 ***
AM-0.25/Doxepin	98.47±9.48	384.53±17.47 *** †
AM-0.5/Doxepin	95.64±8.73	367.86±18.22 *** †
AM-1/Doxepin	101.25±9.79	481.07±17.07 ***

Significant differences between stress and non stress groups for each respective treatment are denoted by *** (*P*< 0.001). In swim stress groups, the significant difference between doxepin, AM-0.25/doxepin, AM-0.5/doxepin and other swim stress groups are demonstrated by † (*P*< 0.05). Data are presented as mean ± SEM (n= 6 rats/group).

(AM-0.25: AM251 (0.25 mg/kg), AM-0.5: AM251 (0.5mg/kg), AM-1: AM251 (1 mg/kg)

(AM...../Doxepin: administration of AM251 prior to the injection of doxepin).

**Table 2.** Effect of the chronic administration of doxepin (10 mg/kg) and AM251 pretreatment on brain regional levels of endocannabinoids.

Endocannabinoid contents following the 28-day treatment period	Brain region	Control	Doxepin	AM-0.25/Doxepin	AM-0. 5/Doxepin	AM-1/Doxepin
AEA (pmol/g tissue)	Prefrontal cortex	9.31±0.57	12.53±0.92*	11.87±0.95*	12.06±0.89*	9.97±0.77
	Hippocampus	21.41±1.56	26.99±1.71*	27.13±1.65*	26.78±1.73*	21.69±1.57
	Hypothalamus	2.38±0.29	4.71±0.42 **	4.35±0.37*	4.86±0.46*	3.11±0.38
	Amygdala	7.56±0.68	11.51±1.31*	11.63±1.29*	10.79±1.16*	8.18±0.63
2-AG (nmol/g tissue)	Prefrontal cortex	3.97±0.31	6.18±0.61**	5.87±0.52 *	6.09±0.59*	4.79±0.46
	Hippocampus	7.95±0.58	10.46±0.62 *	10.58±0.64*	11.21±0.73*	8.46±0.68
	Hypothalamus	7.75±0.77	10.79±0.78 *	10.43±0.73*	9.86±0.64*	7.87±0.70
Amygdala	6.99±0.59	9.26±0.75 *	9.34±0.65*	9.15±0.69*	8.13±0.66

Data were obtained 1h after the last injection and presented as mean±SEM (n=6 rats/group).

(AEA: anandamide, 2-AG: 2-arachidonylglycerol), * P< 0.05, ** P< 0.01.

## Discussion

TCAs are a class of psychotropic medications which are primarily used for the treatment of depression. Attenuation of the stress-induced HPA axis activity and normalization of glucocorticoid feedback and hypersecretion is a common functional response to the chronic treatment with TCAs. According to our findings, chronic treatment with doxepin (10 mg/kg) led to a significant reduction in stress-induced increase in plasma corticosterone level (Table 1). This findings support the notion that a functional response to the chronic treatment with the TCA doxepin, and perhaps other antidepressants, is the attenuation of stress-induced activation of the HPA axis. As previously mentioned, the mechanism(s) by which antidepressants including TCAs regulate the HPA axis activity are not well understood. In recent years, neurobiologists have increasingly turned their attention towards the eCBs as an important brain modulatory system which is able to influence a plethora of physiological functions including the emotional homeostasis and synaptic plasticity (6-9). In addition, the eCBs appears to be involved in the treatment of mood disorders (10). Given the role of the HPA axis in depression, it is interesting to note that the eCBs plays a critical role in the regulation of the HPA axis activity (17-19). In this context, we examined the impact of the pharmacological blockade of the CB_1_ receptors on the peak hormonal response to stress following antidepressant treatment. As shown inTable 1, pre-treatment with AM251 prevented doxepin-induced reduction of plasma corticosterone level in a dose-dependent fashion, suggesting that the engagement of the eCBs is necessary for doxepin to suppress stress-induced activation of the HPA axis. Furthermore, the hypothesis indicating that the eCBs acts as a buffer against the effects of stress in the brain (34) is supported. As previously reported, genetic deletion of the CB_1_ receptors exacerbates stress-induced activation of the HPA axis, whereas, enhancement of the eCB signaling attenuates the HPA axis activity (18). We also showed that chronic treatment with doxepin at the highest dose tested evoked a significant increase of two major endocannabinoids, anandamide and 2-AG, in all brain areas examined (Table 2). This, was blocked by pre-application of AM251 (Table 2), suggesting the existence of an intrinsic eCB activity which contributes to the antidepressant action of doxepin. As a whole, it appears that an interaction between doxepin and the eCBs occurs in the brain regions involved in the regulation of mood and the HPA axis activity. This may contribute to the clinical efficacy of doxepin. In the preclinical models, facilitation of the eCB neurotransmission evokes both antidepressant and anxiolytic effects (7, 8,13). Furthermore, several pharmacological and somatic antidepressant treatments have been shown to increase the eCB neurotransmission which appears necessary for the neurobiological adaptations elicited by these treatments (6, 11,13). To gain a mechanistical insight into the process by which antidepressants including TCAs elevate the eCB contents, the biochemical pathways controlling the eCB production (9), as well as the inhibition of either metabolism or transport of endocannabinoids may be considered in further investigations. Meanwhile, some inconsistencies exist in the literature indicating no change or decrease of the brain regional contents of endocannabinoids following treatment with antidepressants, antipsychotics, or drugs affecting the reward system (30, 35,36). Although it is possible that the derivatization procedure may artifactually affect the eCB levels, the results in our study were similar to those found in the previous ones using a similar technique or liquid chromatography-mass spectrometry which does not require sample derivatization prior to the analysis (37-39). Furthermore, the contents of both anandamide and 2-AG in vehicle-treated control groups (Table 2) were similar to those previously reported by other investigators (37,38). As a whole, the discrepancies among the researchers considering the brain regional levels of endocannabinoids following treatment with psychotropic medications could derive from the differences in the animal species, dosing regimens, experimental protocols, and the neurochemical targets or molecular processes activated by drugs. 

## Conclusion

Our findings suggest that the eCB/CB_1_ receptor signalling is implicated in the mode of action of the TCA doxepin. Chronic exposure to doxepin upregulates the eCB signalling which in turn results in the suppression of the neuroendocrine stress axis. Therefore, it appears that the eCBs may be integral to the development and maintenance of effective coping strategies to stress and might serve as a suitable target for the development of novel antidepressants. 
